# The effect of urban–rural health insurance integration on consumption of middle-aged and older adult households in rural areas: evidence from China

**DOI:** 10.3389/fpubh.2023.1260571

**Published:** 2023-10-24

**Authors:** Lianjie Wang, Qi Hu

**Affiliations:** ^1^Jiangnan University, Wuxi, China; ^2^Zhejiang University of Finance and Economics, Hangzhou, China

**Keywords:** integration of urban and -rural health insurance, rural household consumption, new development pattern, common prosperity, survival consumption

## Abstract

**Introduction:**

Rural consumption is crucial in promoting economic and social development in China’s economic slowdown. Integrating urban-rural residents’ health insurance has alleviated the financial burden of medical expenses for rural households and boosted rural household consumption. This study examines the effect of integrating urban–rural residents’ health insurance on the consumption patterns of middle-aged and older adult households in rural China. Our research provides a reference basis for improving rural healthcare security and enhancing the sustainable consumption capacity in rural areas.

**Methods:**

We employed a Propensity Score Matching Difference-in-Differences model (PSM-DID) to estimate the association between health insurance integration and household consumption using panel data from the China Health and Retirement Longitudinal Study (CHARLS) from 2013 to 2018. Furthermore, we employed a mediation model to analyze the influencing mechanisms.

**Results:**

Our findings suggested a positive association between health insurance integration and survival consumption as well as total consumption among rural middle-aged and older adult households. The conclusion remained valid after endogeneity treatment, robustness and placebo tests. Furthermore, we found that the impact of health insurance integration is more pronounced for middle-aged, female, and high-income rural residents’ households. Integrating urban–rural residents’ health insurance raised consumption by reducing medical expenses and increasing health and life expectancy.

**Discussion:**

Policymakers should deepen the rural medical and health system reform, improve the convenience of medical services for middle-aged and older adult families in rural areas, and improve the medical and life assistance mechanism for vulnerable groups in rural areas. Additionally, the local government should continuously broaden rural household income channels to promote upgrading consumption structure while improving overall consumption levels.

## Introduction

The report of the 20th Party Congress highlighted the importance of building a unified national market, enhancing the reliability of domestic circulation, and strengthening the fundamental role of consumption in economic development (1). Consumption, investment, and exports have been the driving forces behind China’s economic growth. However, the recent cycle of the COVID-19 pandemic has cast a new layer of gloom over international investment and exports. As a result, expanding domestic demand and promoting consumption have become essential for China’s future economic growth. The Fifth Plenary Session of the Nineteenth Central Committee highlighted the need to combine residential consumption with modern production methods and foster a complete domestic demand system in October 2020 (2). Unsurprisingly, the 14th Five-Year Plan once again emphasized the need to accelerate the formation of a new development pattern in which the domestic cycle is the mainstay, and the domestic and international processes promote each other to inject new momentum into economic growth (3). In the “internal circulation and promoting consumption” pattern, rural household consumption is expected to contribute to China’s new economic growth. Statistical data from the National Bureau of Statistics from 2014 to 2020 showed that after deducting the influence of price factors, the annual growth rate of rural household consumption exceeded 6%, which was higher than that of urban areas. This highlights the significant consumption potential in rural areas and is expected to become a crucial driving force for stable economic growth. Despite the significant consumption potential in rural areas, the “dualistic” economic structure between urban and rural areas has resulted in low consumption levels and an unreasonable consumption structure among rural households. The problem of low consumption levels and an unreasonable consumption structure among rural households has persisted in China for a long time. This issue hinders the improvement of living standards and fails to meet the objective requirements of integrated urban–rural economic and social development.

Transforming rural households’ consumption potential into a consumption booster requires overcoming objective barriers, such as health shocks from disease risk that can erode human capital and increased individual precautionary savings to cope with future uncertainty. Lower income levels and higher rates of illness in rural middle-aged and older adult households result in a lack of capacity to cope with health risk shocks. This lack of basic medical coverage inevitably leads to increased precautionary savings and reduced consumption expectations. Establishing and improving the basic medical insurance system and reducing the financial burden of medical care for middle-aged and older adult rural families are crucial to boosting rural consumption in China. The Chinese government established the New Rural Cooperative health System in 2003 to reduce future uncertainty and increase consumption decisions by providing higher levels of protection for rural households. China’s three primary health insurance systems segmented by region, urban–rural areas, and population have resulted in unequal utilization of medical services between urban–rural residents and created complex problems in cross-province settlement, medical treatment in different places, and reimbursement management due to the non-portability of medical insurance benefits. In January 2016, the State Council of China issued the “Opinions on Integrating the Primary Health Insurance System for Urban and Rural Residents,” which proposed the basic principles and specific requirements for integrating the medical insurance system for urban and rural residents (4). Integrating urban rural residents’ health insurance in China follows the basic requirements of higher reimbursement coverage, a more comprehensive health insurance catalog, and more designated hospitals, which has improved medical insurance treatment, reimbursement, and the scope of designated hospitals for rural residents. This has created conditions to protect against health risks, reduce the financial burden of medical treatment, and increase consumption decisions.

The policy of integrating health insurance for urban and rural residents has drawn significant attention from domestic academics due to its implementation and coordination in several provinces. Previous research can be divided into two categories. The first category has examined the direct effects of integrating health insurance for urban and rural residents, such as reducing the medical burden on rural residents (5), enhancing the health of middle-aged and older adult rural populations (6, 7), and mitigating health disparities (8, 9). The second research category of has examined the indirect effects of integrating health insurance for urban and rural residents. These studies found that such integration reduced the likelihood of rural residents falling into poverty due to illness (10, 11), delayed the growth of commercial health insurance (12), and enhanced rural residents’ willingness to migrate to cities (13). In fact, “internal circulation and consumption promotion” has become a crucial aspect of economic development in China’s modernization process. As such, the economic impacts of integrating health insurance for urban and rural residents have emerged as a promising area for future research (14). Whether integrating health insurance for urban and rural residents leads to consumption spillovers is still being determined. While health insurance systems can help individuals manage the risk of future illnesses, they may also decrease the reserve funds available for unexpected events and impact consumption decisions (15, 16). It is important to note that rural health insurance primarily affects subsistence consumption, such as food, due to the long-standing “dualistic” economic structure between rural and urban areas (17). Examining whether institutional integration promotes developmental consumption and upgrades consumption structures could have significant implications for achieving shared prosperity.

This study has several potential contributions. Firstly, While previous research has predominantly focused on rural residents when examining the integration of health insurance for urban–rural residents, there has been limited investigation into the consumption patterns of older adult households in rural areas. Given that middle-aged and older adult households are more susceptible to health and economic risks, they may be particularly affected by health insurance policy reforms. This study systematically investigates whether integrating health insurance for urban and rural residents has a positive impact on consumption among rural middle-aged and older adult households. Secondly, previous research has primarily focused on reducing the financial burden of medical expenses and has yet to thoroughly explore the long-term consequences of health insurance system reforms on household consumption. This paper examines the impact of medical insurance system reforms on household consumption from two perspectives: reducing the financial burden of medical expenses and improving health expectations, and comprehensively assesses both short-term and long-term effects. Thirdly, our study combines the Difference-in-Difference (DID) method and Propensity Score Matching (PSM) to control for the influence of unobservable factors that individual characteristics and changes over time may cause. Furthermore, we test the model settings using multiple methods to ensure the accuracy of the estimation results.

## Theoretical mechanism and research hypothesis

### Financial burden of health care and household consumption

Keynes introduced the theory of consumption function in his book “The General Theory of Employment, Interest, and Money”(1936) (18). This theory posits that total consumption is a function of total income and laid the foundation for research on the consumption function model. The theory of consumption function posits that the level of consumption depends on three factors: income level, objective environmental factors, and subjective factors. However, objective and subjective environmental factors are relatively stable in the short term and can be considered given quantities. This suggests that consumption is a regular function of current income. The theory of consumption function is a fundamental concept in modern economics as it explains the general trend of current consumption. In rural areas, the high burden of medical expenses has long been a significant factor that limiting the income level increase for older adult households. High medical expenses can lead to a decline in the quality of life by reducing other dimensions of consumption expenditure. This is particularly true for low-income rural families, as medical expenses can consume a significant portion of daily costs, such as food, further straining household finances (19). Implementing the New Rural Cooperative Medical Scheme in rural areas in 2003 led to improvements in the utilization rate of medical services, reduced medical and economic risks, and decreased out-of-pocket medical expenses (20, 21). The new rural insurance scheme efficiently induces moral hazard, but its internal logical relationship with the life welfare of middle-aged and older adult is difficult to understand (22). China is striving to achieve common prosperity, and improving the fairness of the social security system is essential to meet the expected economic and social development goals. By integrating urban and rural resident health insurance, implementing six basic requirements has improved medical security treatment, reimbursement of medical expenses, and designated hospital coverage for rural residents, alleviating concerns about medical risks (23). Additionally, the integration has reduced the economic burden of medical treatment for rural residents (9), increasing disposable income for non-medical expenses and creating opportunities for non-medical consumption expenditures.

### Health expectations and household consumption

According to the theory of precautionary saving proposed by Leland (24), individuals tend to increase their savings when facing a more significant risk of future uncertainty, impacting their consumption decisions. The theory of precautionary savings is an innovative model that incorporates uncertainty and analyzes consumer behavior logic regarding consumption in different periods. According to this theory, when future risk increases, the marginal effect of expected future consumption increases, motivating consumers to invest in precautionary savings by allocating more income toward future consumption. Building upon the theory of precautionary savings, Hall proposed the random walk hypothesis in 1978. This hypothesis applies the rational expectations approach to the consumer behavior theory to determine the underlying relationship between current and future consumption (25). Further research suggests that disease risk is a crucial motivator for precautionary savings. Health insurance can help individuals deal with future uncertain disease risks, reducing the need for emergency funds and facilitating consumption (15, 26). This conclusion applies to the older adult population as well, as noted by Clark and Mitchell (27). For a significant period, rural residents in China have commonly adhered to psychological beliefs such as avoiding seeking medical treatment for minor illnesses and delaying treatment for major ones. As a result, they have relied on increasing their precautionary savings to cope with future uncertain medical risks (28). Even though the New Rural Cooperative health Scheme has resulted in higher non-medical consumption expenditures (17, 29), its low level of protection means that it cannot fully address the issue of medical service utilization for vulnerable groups, including low-income individuals and older adults (30, 31). Integrating the urban–rural residents’ health insurance systems has improved the accessibility and health outcomes of medical services for rural residents (32). This improvement is beneficial for reducing future uncertain risks and expanding health expectations, facilitating consumption decisions.

### Research hypotheses

Campbell and Mankiw developed the λ hypothesis (33) by conducting a comprehensive analysis of consumption. This hypothesis categorizes consumption into two types: the first type is determined by the current income level and falls under the Keynesian framework. In contrast, the second type adheres to the random walk hypothesis and is determined by persistent income. By integrating the theoretical analyses presented above, this paper asserts that integrating urban–rural residents’ health insurance has a twofold impact on the consumption of middle-aged and older adult rural households. Firstly, in comparison to the New Rural Cooperative Health Scheme, participation in the integrated insurance plan enables middle-aged and older adult rural households to expand the scope of reimbursable medical services, reduce the economic burden of medical expenses (namely, the self-payment ratio), and increase their disposable income, thereby enhancing their overall consumption level. Secondly, from a long-term perspective, integrating urban–rural residents’ health insurance has improved the medical treatment available to middle-aged and older adult rural households, increased their life expectancy, reduced the need for precautionary savings, and ultimately enhanced consumer confidence. Building on this, the present paper utilizes the consumption function theoretical model and expands upon it as a basic theoretical model for understanding the factors that influence consumption. This paper examines the impact of integrating urban–rural residents’ health insurance on the consumption of middle-aged and older adult rural households from the “current” perspective and the “long-term” perspective while assuming that other factors remain constant. It should be noted that, due to the constraints of rural economic development and the impact of agricultural production activities, the income level of middle-aged and older adult rural households has traditionally been lower than that of their urban counterparts, and their sources of income are relatively limited. Middle-aged and older adult rural households prioritize survival consumption, such as food, clothing, and fuel, Andover-development consumption, including tourism, heating, education, and healthcare, resulting in low consumption levels and an imbalanced consumption structure. By reducing the medical burden on these households, integrating urban–rural health insurance can increase their health expectations and potentially positively affect subsistence consumption. Furthermore, integrating urban–rural residents’ health insurance leads to a shift in consumption priorities, emphasizing development consumption more than survival consumption. In that case, it may have a positive spillover effect on overall consumption. The study’s potential theoretical contribution is integrating rational expectations from the random walk hypothesis and uncertainty expectations from the precautionary savings theory into the analytical framework. This enriches and extends the theoretical model of the consumption function, laying the groundwork for a comprehensive examination of the policy effects of integrating urban–rural residents’ health insurance.

Based on the above discussion, this paper proposes the following research hypotheses:

*Hypothesis 1*: integrating urban–rural residents’ health insurance benefits middle-aged and older adult rural households by increasing their survival, development, and total consumption.

*Hypothesis 2*: integrating urban–rural residents’ health insurance increases the consumption of middle-aged and older adult rural households by reducing the economic burden of healthcare costs.

*Hypothesis 3*: integrating urban–rural residents’ health insurance increases the consumption of middle-aged and older adult rural households by improving their expectations for future health outcomes.

## Data, variables, and methods

### Data source

This article utilizes data from the China Health and Aging Tracking Survey (CHARLS), a large-scale interdisciplinary survey conducted by the National Development Research Institute of Peking University in collaboration with the China Social Survey Research Centre and the Peking University Youth League Committee. This survey gathered high-quality micro-data representative of Chinese middle-aged and older adult households and individuals, with approximately 20,000 respondents from 28 provinces, autonomous regions, and municipalities directly under the Central Government. This article utilizes CHARLS data for two primary reasons. Firstly, the survey questionnaire design draws on international experience from countries such as the United States and Europe and employs various sampling methods such as PPS. The questionnaire covers essential information on individuals and household structures, health insurance and healthcare utilization, and multiple types of income and expenditure structures. These features provide high-quality data support for investigating rural household consumption issues. Secondly, the four surveys cover the implementation cycle of the policy of integrating health insurance for urban and rural residents. The data set includes variables related to the New Rural Cooperative and basic health insurance for urban and rural residents, which are more suitable for the research needs of this paper. This study utilizes the two-period treatment of 2013 and 2018 to obtain balanced panel data, given that the initial survey in 2011 lacked a straightforward questionnaire design. The research population consists of rural middle-aged and older adult households. Only complete two-period data samples of participants in the New Rural Cooperative Scheme and urban–rural residents’ health insurance were retained, resulting in a final sample size of 12,378, including 1,690 samples in the treatment group and 10,688 in the control group.

### Variables

The dependent variable studied in this articles household consumption. This paper has established three indicators based on the questionnaire design and research needs. ①Survival consumption reflects the necessary consumption expenditures for rural older adult households to maintain their daily lives. To calculate the annual survival consumption, the expenses on food, clothing, communication, water, electricity, and fuel in the past month are multiplied by 12 months. ②Developmental consumption, reflects the expenditures made by rural older adult households to improve and enhance their quality of life. To calculate the total developmental consumption, the expenditures on tourism, heating, consumer goods and appliances, education and training, healthcare, beauty, and automobiles in the past year are added. ③The third indicator, Total Consumption, reflects the overall consumption situation of rural households. To calculate the total annual consumption of rural older adult households, the Survival and Developmental Consumption are added. Additionally, to eliminate the influence of changes in the price level, all consumption indicators in this paper were adjusted using the current year’s CPI index. The dependent variables were transformed using the natural logarithm to reduce research bias, resulting in three variables: Ln_Survival for Survival Consumption, Ln_Developmental for Developmental Consumption, and Ln_Total for Total Consumption.

The explanatory variable under study in this article is Insur_Integration, which integrates the health insurance of urban and rural residents. This variable is obtained by processing the different types of medical insurance (Med_Insurance) in the questionnaire. To ensure accurate measurement, sample data from provinces that completed system integration as early as 2008 were excluded from the 2013 dataset. Only samples from participants in the New Rural Cooperative Medical Scheme were included. The previous DID model defined two groups of dummy variables as follows: policy dummy variables included those who participated in the New Agricultural Cooperative Scheme in 2013 and those who participated in urban and rural residents’ insurance in 2018, assigned a value of 1 as the treatment group while those who participated in the New Agricultural Cooperative Scheme in both 2013 and 2018 were assigned a value of 0 as the control group Additionally, the time dummy variable (Time) was assigned a value of 0 in 2013 and 1 in 2018, with 2016 as the policy implementation point.

In order to control for the influence of other variables on the consumption of households in rural areas, this study refers to the research conducted by Ma et al. (17) and has selected individual characteristics, income levels, and lifestyles as control variables. We selected these factors for several reasons. Firstly, according to Keynesian consumption theory, income is considered a fundamental factor influencing consumption and therefore must be included as a control variable. Secondly, individual characteristics and lifestyle factors play a significant role in influencing the consumption patterns of older adult families, impacting their health conditions, consumption habits, and preferences. These variables included age, gender, marital status, education level, the logarithm of annual household income (Ln_Income), smoking, drinking, and social activities. The logarithm of annual household income (Ln_Income) is calculated by summing up five different sources of income, including wage income, agricultural income, social security transfer income, personal income, and government subsidy income, and then taking the logarithm. Sociability is defined as participation in any social activity in the past month, based on an 11-item questionnaire. A value of 1 is assigned if the respondent participated in any social activity and a value of 0 if they did not.

Based on the previous analysis, two types of variables in this study affect the mechanism. Firstly, the integration of urban and rural resident medical insurance reduces the out-of-pocket ratio of health expenses for rural older adult households, thereby easing the economic burden of medical treatment. The primary measurement variable used is the “Proportion of medical expenses to total expenses” of rural residents in the CHARLS questionnaire, which is obtained by dividing medical expenses by total expenses. The second variable type involves rural residents’ improvement in future health and life expectations. The leading indicators for measurement are “health expectancy” and “future hope” as answered by rural residents in the questionnaire. Respondents’ answers to “Health_Expectancy” were scored 1–5 based on “almost impossible, unlikely, possible, very likely, and definitely, “respectively, with higher scores indicating better future health expectancy.” For “Future_Hope,” respondents’ answers of “very little or none, not much, sometimes or average time, most of the time” were scored 1–4, with higher scores indicating better life expectancy.

## Methods

The gradual transition from the new rural cooperative to basic medical insurance for both urban and rural residents creates a quasi-natural experimental setting to investigate the impact of integrating medical insurance on the consumption patterns of middle-aged and older adult rural households. This study employs a double-difference-in-differences (DID) model to mitigate endogeneity issues arising from omitted variables or selectivity bias. Two sets of dummy variables are constructed to conduct the DID analysis: ① Policy dummy, comprising a treatment group affected by the policy and a control group not affected by the policy.② Time dummy, including two periods before and after policy implementation. By comparing the consumption differences between the two sample groups before and after the policy implementation, the study systematically evaluates the impact mechanism of integrating medical insurance on the consumption patterns of urban and rural residents. We constructed a basic DID model, as illustrated in Equation (1):

(1) $$C_{\mathrm{it}}=\beta_{0}+\beta_{1}du+\beta_{2}dt+\beta_{3}du\times dt+\beta_{4}\mathrm{control}+\varepsilon_{\mathrm{it}}$$

Where $C_{\mathrm{it}}$ represents the consumption status of the *i-th* sample in period *t*; du is the policy dummy variable, assigned a value of 1 for the treatment group and 0 for the control group dt is the time dummy variable, assigned a value of 0 before policy implementation and 1 after policy implementation. The interaction term between policy and time dummy variables is represented as du×dt , control variables are denoted as control, $\varepsilon_{\mathrm{it}}$ is the error term, $\beta_{0}$ is the intercept, and, $\beta_{1}$ , $\beta_{2}$ , $\beta_{3}$ , $\beta_{4}$ are the corresponding regression coefficients. The net effect of integrating urban and rural residents’ medical insurance on consumption, represented by the regression coefficient $\beta_{3}$ , was evaluated using the DID model with control for the policy and time dummy variables in the treatment group. The results are presented in Table 1.

**Table 1:** The effects of the treatment group and the control group.

	**Before the policy implementation**	**After the policy implementation**	**Difference**
**Treatment group**	$\beta_{0}+\beta_{1}$	$\beta_{0}+\beta_{1}+\beta_{2}+\beta_{3}$	$\beta_{2}+\beta_{3}$
**Control group**	$\beta_{0}$	$\beta_{0}+\beta_{2}$	$\beta_{2}$
**Difference**	$\beta_{1}$	$\beta_{1}+\beta_{3}$	$\beta_{3}$ (D-in-D)

The DID model is based on a natural experiment where the treatment and control groups are randomly assigned, meeting the requirements of a natural experiment. This study aims to estimate the average treatment effect using the formula presented in Equation (2):

(2) $$\begin{array}{l} \;ATT=E\left\{C_{i}\left(1\right)-C_{i}\left(0\right)|du=1\right\}=E\left\{C_{i}\left(1\right)|du=1\right\}-\\ E\left\{C_{i}\left(0\right)|du=1\right\} \end{array}$$

In fact, we cannot observe the consumption status of the treatment group before the policy implementation, i.e., $E\left\{C_{i}\left(0\right)|du=1\right\}$ is a counterfactual estimator. Instead, we use the observed results of sample i under the non-intervention state, $E\left\{C_{i}\left(0\right)|du=0\right\}$ , as a substitute to estimate the potential results of individuals under intervention. Based on this, equation (2) can be further expressed as:

(3) $$\begin{array}{l} ATT=\left(E\left\{C_{i}\left(1\right)|du=1\right\}-E\left\{C_{i}\left(0\right)|du=1\right\}\right)+\\ \left(E\left\{C_{i}\left(0\right)|du=1\right\}-E\left\{C_{i}\left(0\right)|du=0\right\}\right)\\ =ATT+\mathrm{SelectionBias} \end{array}$$

The average net treatment effect (ATT) of integrating urban–rural health insurance on consumption is determined, with ATT > 0 indicating a positive result and vice versa indicating a negative effect. Nevertheless, compared to an ideal experiment, the integration of rural middle-aged and older adult households into urban and rural residents’ health insurance is affected by various factors that make it challenging to ensure consistency in the relevant characteristics. This study utilizes the Propensity Score Matching-Difference-in-Differences (PSM-DID) model to validate the findings. This model controls for covariates and matches the treatment group and control group based on similar or identical scores, eliminating selection bias and providing a more accurate evaluation of the policy effects of integrating urban and rural resident medical insurance.

(4) $$C_{\mathrm{it}}^{PSM}=\beta_{0}+\beta_{1}du+\beta_{2}dt+\beta_{3}du\times dt+\beta_{4}\mathrm{control}+\varepsilon_{\mathrm{it}}$$

## Results

### Descriptive statistical analysis

Table 2 presents the descriptive statistics of the main variables in this paper. The average annual expenditures on survival, developmental, and total consumption for middle-aged and older adult households in rural areas are 14,032.71 yuan, 11,327 yuan, and 25,404.02 yuan. These values are 8.761, 8.445, and 9.455 after logarithmic transformation, respectively. The logarithmic means of the three consumption groups in the treatment group sample are higher than those in the control group, indicating an improvement in the consumption level of rural older adult households after integrating urban–rural health insurance. The independent variables include a 7.5% proportion of samples participating in basic health insurance for urban and rural residents and a 92.5% proportion of samples participating in the new rural cooperative health system. The control variables consist of an average age of approximately 62.299 for rural older adult, a slightly higher proportion of male residents at 51.3%, and a majority of rural older adults having spouses, accounting for about 78.9%. The average years of education for the sample population are less than 5 years. The average annual total income for older adult households in rural areas is 21,402.41 yuan. The logarithm of total household income is 8.224, slightly lower than the total consumption, which the lower rural household income may influence in 2013. The proportion of rural residents who smoke and drink is 26.5 and 35.4%, respectively. More than 50% of rural older adults participate in social activities. The proportion of medical expenses to total consumption is approximately 17.7%. The mean healthy life expectancy and future hope scores are 2.917 and 2.538, respectively. More than half of rural older adults have positive expectations for healthy life expectancy and future hope, with proportions of 63.65 and 54.42%, respectively. However, some older adults remain concerned about their health and future.

**Table 2:** Descriptive statistical results.

Variables	Definition	Full sample		Treatment group		Control group	
		Mean	SD	Mean	SD	Mean	SD
*Ln_Survival*	Log of survival consumption	8.761	1.160	8.834	1.197	8.749	1.153
*Ln_ Developmental*	Log of developmental consumption	8.445	1.481	8.518	1.509	8.433	1.476
*Ln_Total*	Log of total consumption	9.455	1.156	9.530	1.213	9.442	1.146
*Med_Insurance*	New Agricultural Cooperative = 0, Urban and Rural Health Insurance = 1	0.075	0.263	0.500	0.500	0.000	0.000
*Age*	Average age	62.299	9.556	62.343	9.574	62.292	9.553
*Gender*	Female = 0, Male = 1	0.513	0.500	0.525	0.500	0.510	0.500
*Marital status*	No = 0, with spouse = 1	0.789	0.408	0.793	0.405	0.788	0.409
*Education level*	Years of education	4.785	3.793	4.852	3.744	4.774	3.802
*Ln_Income*	Log of total household income	8.224	2.094	8.350	2.132	8.203	2.087
*Smoking*	Non-smoking = 0, smoking = 1	0.265	0.441	0.246	0.431	0.268	0.443
*Drinking*	No alcohol = 0, alcohol = 1	0.354	0.478	0.374	0.484	0.350	0.477
*Social activities*	Not participating = 0, participating = 1	0.527	0.499	0.496	0.500	0.532	0.499
*Proportion*	Proportion of medical consumption in total consumption	0.177	1.184	0.168	0.734	0.227	2.559
*Health_Expectancy*	Health expectations	2.917	1.264	3.037	1.247	2.896	1.266
*Future_Hope*	Hopeful for the future	2.538	1.282	2.536	1.290	2.539	1.281

Note: Decimal places are rounded to three digits.

### Main empirical results

Table 3 presents the results of a stepwise regression analysis conducted to investigate the impact of integrating urban and rural residents’ health insurance on the spending behavior of middle-aged and older adult rural households. Models (1) to (3) were estimated without controlling for any other factors, while models (4) to (6) included additional control variables. Models (1) and (4) indicate a significant positive effect of integrating urban–rural health insurance on the survival consumption of rural middle-aged and older adult households. Models (3) and (6) indicate a significant positive effect of integrating urban–rural health insurance on the total consumption of rural middle-aged and older adult households. However, models (2) and (5) suggest that the integration of urban and rural health insurance does not significantly impact on the developmental consumption. In summary, integrating urban–rural health insurance has led to increased consumption among rural middle-aged and older adult households, particularly in survival consumption. Supporting research hypothesis 1 partially. Control variables, including Age, Marital status, Education level, Ln_income, Smoking, and Drinking, have impacted the consumption of rural middle-aged and older adult households. The regression results for each variable were generally consistent with the predictions of consumption economics.

**Table 3:** Empirical regression results.

Variables	(1)	(2)	(3)	(4)	(5)	(6)
	*Ln_Survival*	*Ln_ Developmental*	*Ln_Total*	*Ln_Survival*	*Ln_ Developmental*	*Ln_Total*
*DID*	0.086** (0.035)	0.089 (0.054)	0.087** (0.037)	0.073** (0.063)	−0.018 (0.101)	0.070** (0.072)
*Policy*	0.006 (0.045)	0.065 (0.064)	0.035 (0.052)	−0.029 (0.046)	0.010 (0.068)	−0.005 (0.054)
*Time*	1.178*** (0.018)	0.099*** (0.026)	0.784*** (0.019)	1.106*** (0.032)	0.020 (0.048)	0.720*** (0.034)
*Age*				−0.017*** (0.002)	−0.022*** (0.003)	−0.020*** (0.002)
*Gender*				−0.048 (0.040)	−0.038 (0.060)	−0.043 (0.045)
*Marital status*				0.186*** (0.034)	0.167*** (0.051)	0.173*** (0.038)
*Education level*				0.025*** (0.004)	0.032*** (0.006)	0.029*** (0.004)
*Ln_Income*				0.098*** (0.007)	0.124*** (0.011)	0.111*** (0.009)
*Smoking*				−0.100*** (0.038)	−0.098* (0.059)	−0.109** (0.045)
*Drinking*				−0.008 (0.030)	−0.150*** (0.046)	−0.073** (0.034)
*Social activities*				0.024 (0.025)	0.020 (0.039)	0.011 (0.028)
*Province*	YES	YES	YES	YES	YES	YES
*_Cons*	8.076*** (0.015)	8.379*** (0.022)	8.996*** (0.017)	8.124*** (0.126)	8.534*** (0.197)	9.112*** (0.143)
*N*	10,500	10,264	10,523	9,816	9,682	9,820

Note: * *p* < 0.1, ** *p* < 0.05, *** *p* < 0.01.

### Robustness test

**Figure 1:**
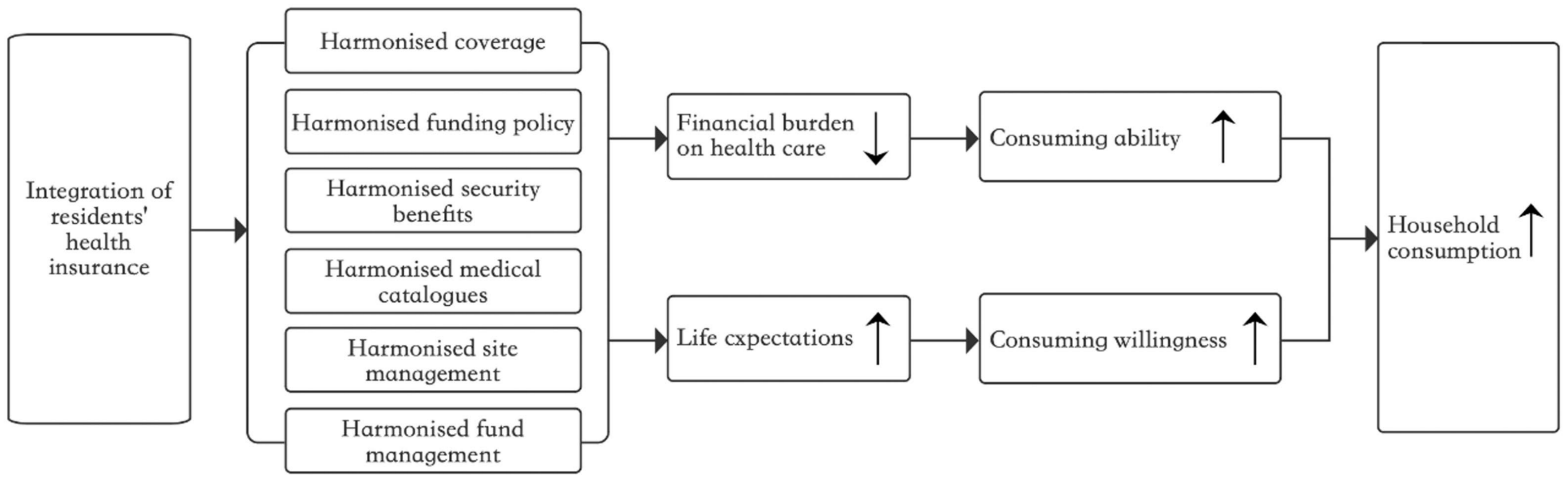
Mechanisms through which the integration of health insurance affects household consumption.

**Figure 2:**
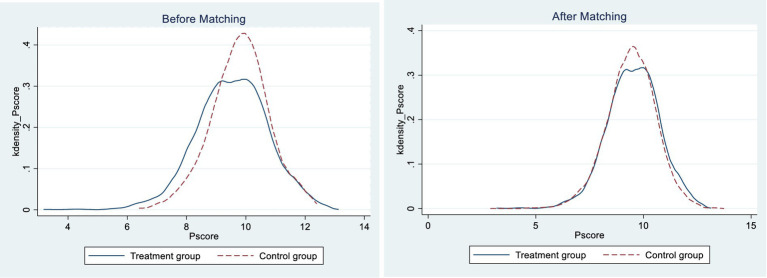
Kernel density plots for the treatment and control group.

The empirical findings indicate that integrating health insurance for urban–rural residents improves the survival and total consumption of rural middle-aged and older adult households. Nonetheless, it should be noted that the decision of rural residents to participate in the urban and rural residents’ basic medical insurance program is not entirely random and is subject to various influencing factors. Consequently, it poses a challenge to ensure the similarity of fundamental characteristics in the standard DID method, and there could be significant variations between the basic characteristics of rural middle-aged and older adult residents in the treatment and control groups. This discrepancy may lead to the regression model not satisfying the assumptions of random selection, resulting in endogeneity issues. The PSM-DID model was subsequently validated. Using total consumption as an example, it is evident from the plots that there is a substantial difference in propensity score values between the treatment and control groups before and after matching (Figure 1). This observation implies that the sample was subject to selection bias before propensity score matching and direct inclusion in the regression analysis may have led to biased results. The kernel density plot after matching reveals that the propensity score distributions of the treatment and control groups are nearly overlapping. The results indicate that after matching the two groups, sample selection bias is significantly reduced, aligning with the assumption of a randomized experiment (Figure 2).

**Figure 3:**
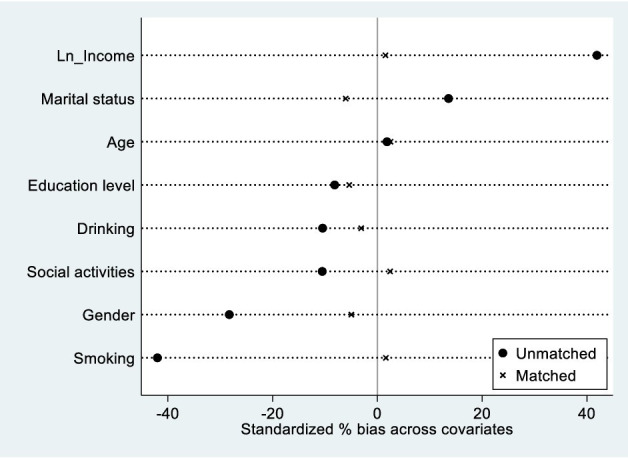
Variables’ normalization deviation.

**Figure 4:**
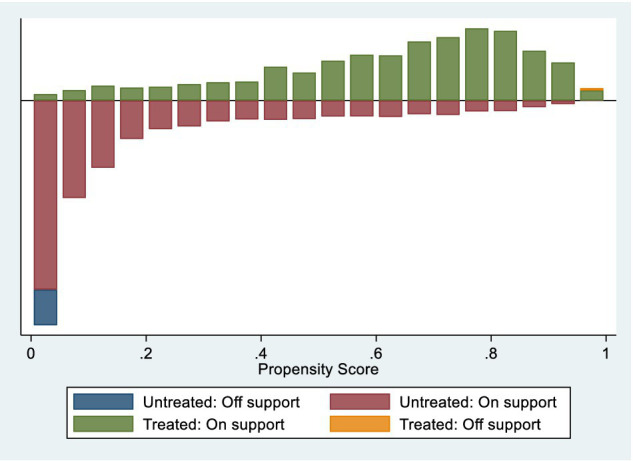
PSM common value ranges.

Figures 3, 4 present the variables’ normalization deviations and common value ranges. The descriptive statistics and balance tests for covariates are presented in Table 4. As expected, before matching, the treatment group sample exhibited several characteristics that differed from those of the control group. Specifically, the treatment group had a higher proportion of female residents, a higher proportion of residents with spouses, a lower level of education, a higher level of income, and a lower proportion of individuals who smoked, drank, and participated in social activities. Additionally, the treatment group was older than the control group. Furthermore, the t-test results indicated significant bias in all covariates between the control and treatment groups before matching. Therefore, propensity score matching was necessary to eliminate selectivity bias. The equilibrium test results indicated that systematic deviations in all variables between the treatment and control groups were significantly reduced after matching. The absolute values of the t-tests were below 0.6, indicating that significant differences in covariate characteristics between the two groups were eliminated. The conditions of the equilibrium hypothesis were satisfied. Furthermore, after propensity score matching, all samples in the treatment group were within the common range of values, while 495 samples in the control group were outside this range. Only a small proportion of samples (approximately 4%) were lost due to propensity score matching. In summary, PSM-DID reduces systematic bias in sample characteristics, resulting in more robust estimation results.

**Table 4:** Balance test.

Variables	Before matching				After matching			
	Control	Treat	Diff	|t|	Control	Treat	Diff	|t|
*Age*	60.470	63.712	3.242***	6.29	63.548	63.712	0.164	0.23
*Gender*	0.405	0.220	−0.185***	6.66	0.238	0.220	−0.018	0.56
*Marital status*	0.780	0.827	0.047**	1.97	0.839	0.827	−0.012	0.42
*Education level*	4.662	4.012	−0.65***	2.97	4.170	4.012	−0.158	0.52
*Ln_Income*	7.946	9.224	1.278***	11.52	9.233	9.224	−0.009	0.08
*Smoking*	0.295	0.040	−0.255***	9.99	0.040	0.040	0.000	0.00
*Drinking*	0.304	0.207	−0.097***	3.69	0.211	0.207	−0.004	0.10
*Social activities*	0.556	0.480	−0.076***	2.69	0.464	0.480	0.016	0.39

Note: * *p* < 0.1, ** *p* < 0.05, *** *p* < 0.01.

Table 5 presents the results of the PSM-DID estimation. The findings indicate that survival consumption values differ between the pre-policy treatment group and the control group by a smaller amount (−0.013) compared to the post-policy differential (0.050), as shown by the single difference between the 2013 and 2018 samples. The results of the PSM-DID analysis reveal that integrating urban–rural health insurance increased survival consumption among rural middle-aged and older adult households by 6.3%. Additionally, the difference in consumption scores between the treatment and control groups was smaller before policy implementation (0.015) than after implementation (0.081), indicating a significant increase in total consumption among rural middle-aged and older adult households due to integrating urban–rural residents’ health insurance. The results of the PSM-DID analysis indicate that integrating urban–rural health insurance has increased total consumption by 6.6% among rural middle-aged and older adult households.

**Table 5:** Results based on the PSM-DID method.

Samples		(1)	(2)
		Ln_Survival	Ln_Total
2013	Control Group	8.035	8.941
	Treat Group	8.022	8.956
	Diff(T-C)	−0.013* (0.033)	0.015 (0.037)
2018	Control Group	9.214	9.737
	Treat Group	9.264	9.818
	Diff(T-C)	0.050** (0.035)	0.081** (0.038)
PSM-DID		0.063** (0.048)	0.066** (0.053)
N		9,816	9,820
R-square		0.32	0.27

Note: * *p* < 0.1, ** *p* < 0.05, *** *p* < 0.01.

### Placebo test

The placebo test is a standard method for evaluating policy effectiveness by creating a sample of dummy policy implementation times or experimental groups to determine whether the policy effect is genuine. Based on the combined use of PSM-DID in the previous section, it can be concluded that integrating urban and rural health insurance benefits improves subsistence and total consumption. However, it is still necessary to account for the impact of other unobservable factors. Table 6 displays the results of the assessment analysis conducted on two periods of CHARLS data from 2013 and 2015by the research design of this paper. The rationale for selecting these periods was based on the placebo test. Before integrating urban–rural residents’ health insurance, the panel data for the two periods were reconstructed. The regression coefficients for DID in models (1) and (2) are insignificant, indicating that other uncontrollable factors brought about by the evolution over time do not affect the boosting effect of urban–rural health insurance integration on the consumption of rural middle-aged and older adult households. The placebo test further supports the benchmark regression results.

**Table 6:** Placebo test results.

Variables	(1)	(2)
	*Ln_Survival*	*Ln_Total*
*DID*	0.047 (0.032)	−0.017 (0.033)
*Policy*	−0.574 (0.426)	0.285 (0.461)
*Time*	−0.163*** (0.062)	0.311*** (0.066)
Control variables /provinces	YES	YES
*_Cons*	9.979*** (0.841)	9.702*** (0.910)
*N*	9,145	9,238

Note: * *p* < 0.1, ** *p* < 0.05, *** *p* < 0.01.

### Heterogeneity analysis

Life-cycle theory posits a relationship between consumers’ income and the expected value of income at different stages of life, including childhood, middle age, and old age (34). The impact of policy measures may vary due to the progressive decline in working capacity, income levels, and consumption expectations associated with old age. Rural female residents face disadvantages in income sources, labor market values, and health care policy concerns compared to their male counterparts, particularly in comparison to urban areas. These disadvantages accumulate over the life cycle and may result in gender differentiation in policy effects (35). In addition, according to the Keynesian theory of the consumption function, among the many factors that influence residents’ consumption, the level of income and its distribution is the most fundamental factor, i.e., total consumption is a function of total income (36). This paper analyses the heterogeneity of urban and rural residents’ health insurance integration affecting consumption along three dimensions: age (45–59 and 60 and above), gender (male and female), and income distribution (low and high income).

Tables 7, 8 present the results of the heterogeneity analysis, which revealed similar findings for survival consumption and total consumption. The results indicate that system consolidation benefits middle-aged, female, and high-income rural households by increasing their consumption levels. However, the impact on consumption for older adult, male, and low-income rural households is insignificant. This suggests that integrating urban–rural residents’ health insurance has limited effects on the quality of life for families in these groups. The integration of urban and rural residents’ health insurance has improved the accessibility of healthcare services for rural middle-aged and older women, thereby reducing the financial burden of healthcare and promoting increased household consumption expectations.

**Table 7:** Heterogeneity analysis of survival consumption.

Variables	(1)	(2)	(3)	(4)	(5)	(6)
	45–59	60+	Male	Female	Lower income	Higher income
*DID*	0.204*** (0.078)	0.039 (0.065)	−0.037 (0.112)	0.147*** (0.055)	−0.006 (0.070)	0.165*** (0.068)
*Policy*	−0.007 (0.055)	−0.054 (0.074)	−0.035 (0.063)	−0.026 (0.066)	−0.017 (0.063)	−0.034 (0.064)
*Time*	1.069*** (0.042)	1.055*** (0.045)	1.138*** (0.058)	1.095*** (0.038)	1.229*** (0.054)	1.081*** (0.045)
Control variables /provinces	YES	YES	YES	YES	YES	YES
*_Cons*	7.407*** (0.084)	6.644*** (0.102)	8.050*** (0.225)	8.117*** (0.160)	8.716*** (0.165)	6.711*** (0.290)
*N*	4,123	5,693	5,035	4,781	4,692	4,677

Note: * *p* < 0.1, ** *p* < 0.05, *** *p* < 0.01.

**Table 8:** Heterogeneity analysis of total consumption.

Variables	(1)	(2)	(3)	(4)	(5)	(6)
	45–59	60+	Male	Female	Lower income	Higher income
*DID*	0.136** (0.084)	−0.032 (0.069)	0.023 (0.133)	0.084** (0.092)	−0.006 (0.107)	0.065** (0.097)
*Policy*	0.039 (0.068)	−0.046 (0.086)	0.009 (0.003)	−0.021 (0.079)	−0.009 (0.075)	0.012 (0.003)
*Time*	0.701*** (0.046)	0.636*** (0.048)	0.692*** (0.062)	0.718*** (0.042)	0.871*** (0.058)	0.638*** (0.051)
Control variables /provinces	YES	YES	YES	YES	YES	YES
*_Cons*	8.336*** (0.100)	7.357*** (0.111)	9.225*** (0.264)	9.029*** (0.177)	9.846*** (0.191)	7.394*** (0.327)
*N*	4,124	5,696	5,028	4,792	4,910	4,910

Note: * *p* < 0.1, ** *p* < 0.05, *** *p* < 0.01.

### Influence mechanisms analysis

The integration of urban and rural health insurance has the potential to impact rural middle-aged and older adult household consumption through two pathways. Firstly, it can reduce the current medical out-of-pocket ratio for middle-aged and older adult rural households, increasing their relative income and current consumption levels. Second, it can raise the future health expectations of rural residents, leading to greater optimism in coping with future uncertainties and ultimately increasing household consumption intentions. This paper investigates how the integration of urban and rural health insurance affects the consumption of rural middle-aged and older adult households through three mediating variables: “Proportion,” “Health Expectancy” and “Future Hope.” This paper employs the mediating effect test developed by Wen and Ye (37) to analyze the impact mechanism, and constructs a model using equations (5) to (7):

(5) $$C_{\mathrm{it}}=\alpha_{0}+\alpha_{1}DID_{\mathrm{it}}+\alpha_{2}\mathrm{Polic}y_{i}+\alpha_{3}Time_{t}+\alpha_{4}X_{\mathrm{it}}+\varepsilon_{\mathrm{it}}$$

(6) $$M_{\mathrm{it}}=\gamma_{0}+\gamma_{1}DID_{\mathrm{it}}+\gamma_{2}\mathrm{Polic}y_{i}+\gamma_{3}Time_{t}+\gamma_{4}X_{\mathrm{it}}+\varepsilon_{\mathrm{it}}$$

(7) $$C_{\mathrm{it}}=\delta_{0}+\delta_{1}DID_{\mathrm{it}}+\delta_{2}\mathrm{Polic}y_{i}+\delta_{3}Time_{t}+\delta_{4}X_{\mathrm{it}}+\delta_{5}M_{\mathrm{it}}+\varepsilon_{\mathrm{it}}$$

The mediating variable is represented by $M_{\mathrm{it}}$ . Based on equations (5) to (7), the test procedure is as follows: Step 1: The effect of the integration of urban–rural residents’ health insurance on the consumption of rural middle-aged and older adult households is examined using the underlying model. If the regression coefficient is significant, the next step is taken. Step 2: The model includes the mediating variables as the explanatory variable for testing. If the DID coefficient is significant, it indicates the presence of a mediating effect. Step 3: The mediating variable and DID are separately included in the model for testing, and the change in the regression coefficient is examined to determine if the mediating effect holds. As Table 2 demonstrates the benefits of system integration in raising subsistence and total consumption, Step 2 directly assesses the impact of urban and rural health insurance integration on the mediating variables.

Based on this, Step 3 in this paper is to include the three mediating variables of “Proportion,” “Health_Expectancy,” and “Future_Hope” in the regression model for analysis, as shown in Table 9. Therefore, as presented in Table 10, Step 3 involves analyzing the regression model by including the three mediating variables of “Proportion,” “Health Expectancy,” and “Future Hope.” Model (1) indicates that the proportion of medical consumption reduces the survival consumption of rural middle-aged and older adult households, suggesting that medical consumption significantly displaces daily consumption expenditure. Meanwhile, models (2) and (3) demonstrate that “Healthy Expectancy” and “Future Hope” increase survivorship consumption by 10.1 and 2.9%, respectively. The results suggest that rural middle-aged and older adult households with positive expectations for their future health and life tend to increase their consumption. Additionally, models (4) to (6) reveal a significant negative relationship between the proportion of healthcare consumption and the total consumption of rural households. Furthermore, “Health Expectancy” and “Future Hope” increase the total consumption level of rural middle-aged and older adult households. Combining the results of the regression tables, it is clear that all three mediating variables passed the mechanism test; that is, integrating urban–rural residents’ health insurance acts on household consumption by reducing the share of medical consumption and raising future health and life expectations. However, it has a higher impact on survival consumption than total consumption. These findings suggest the positive role that integrating urban–rural health insurance in enhancing the well-being of middle-aged and older adult rural families.

**Table 9:** Impact of medical insurance integration on mediating variables.

Variables	(1)	(2)	(3)
	Proportion	Health_Expectancy	Future_Hope
*DID*	−0.014* (0.032)	0.087** (0.043)	0.074** (0.090)
*Policy*	−0.005 (0.013)	0.225*** (0.061)	−0.008 (0.056)
*Time*	−0.153*** (0.020)	−0.209*** (0.041)	0.181*** (0.041)
Control variables /provinces	YES	YES	YES
*_Cons*	0.060 (0.049)	2.512*** (0.182)	2.795*** (0.164)
*N*	8,820	8,521	8,485

Note: * *p* < 0.1, ** *p* < 0.05, *** *p* < 0.01.

**Table 10:** Results of impact mechanisms analysis.

Variables	(1)	(2)	(3)	(4)	(5)	(6)
	*Ln_Survival*	*Ln_Survival*	*Ln_Survival*	*Ln_Total*	*Ln_Total*	*Ln_Total*
*DID*	0.102* (0.074)	0.061*** (0.019)	0.055*** (0.172)	0.050** (0.072)	0.066 (0.076)	0.070 (0.071)
*Policy*	−0.041 (0.051)	−0.328** (0.165)	−0.267* (0.150)	−0.005 (0.054)	−0.014 (0.057)	−0.018 (0.054)
*Time*	1.088*** (0.036)	2.371*** (0.089)	2.410*** (0.083)	0.719*** (0.035)	0.693*** (0.037)	0.698*** (0.035)
*Proportion*	−0.235*** (0.059)			−0.006*** (0.055)		
*Health_Expectancy*		0.101*** (0.030)			0.025*** (0.012)	
*Future_Hope*			0.029* (0.028)			0.024*** (0.011)
Control variables /provinces	YES	YES	YES	YES	YES	YES
*_Cons*	8.139*** (0.132)	6.594*** (0.394)	6.712*** (0.365)	9.112*** (0.143)	9.110*** (0.162)	9.074*** (0.147)
*N*	8,820	8,499	8,460	8,820	8,776	8,615

Note: * *p* < 0.1, ** *p* < 0.05, *** *p* < 0.01.

## Discussion

This study utilized panel data from the China Health and Retirement Longitudinal Study (CHARLS) for 2013–2018 to analyze the impact of the integration of medical insurance policies on household consumption among middle-aged and older adult in rural areas. Firstly, the study indicates that integrating health insurance for urban–rural residents has increased survival consumption by 7.3% and total consumption by 7.0% in rural households. This finding is consistent with the conclusions of previous studies such as Chen and Zhang (38) and Bai et al. (29). For a long time, rural households have had lower income levels compared to urban areas. They often rely on a single source of income, and their ability to cope with the risks and shocks of illnesses is generally inadequate. The integration of health insurance for urban–rural residents has expanded the accessibility of healthcare services for older adult households in rural areas, reducing the economic burden of medical expenses. This integration is beneficial as it increases the relative income, thus releasing the demand for daily living expenses. However, its impact on consumption in tourism and healthcare could be more substantial. This objectively reflects the limited consumption crowding-in effect of integrating health insurance for urban and rural residents, primarily focused on essential consumption. There is still significant potential for increased consumption among middle-aged and older adult residents in rural areas. Sun et al. (39) have argued that the government should combine efforts to enhance the fairness of the social security system with expanding income channels and rural industrial transformation. That will lay the foundation for improving the living standards of rural residents.

The heterogeneous results of this study indicate that the integration of medical insurance for urban and rural residents has similar effects on the heterogeneity of survival consumption and total consumption. Institutional integration is beneficial for increasing the consumption levels of middle-aged individuals, women, and high-income households in rural areas. In contrast, its impact on the consumption of older adult individuals, men, and low-income rural households is not significant. This study suggests that the possible reason for these results is that although the integration of integrating health insurance for urban and rural residents reduces medical costs for older adult and low-income rural residents, their income uncertainty and medical economic risks remain high. As a result, their household consumption decisions become more cautious (40). In addition, Chinese female residents generally have a higher life expectancy than males. However, most of them live with chronic illnesses, experiencing a higher probability of disease risks than male residents. They also bear a heavier burden of medical expenses (41). Cheng et al. (42) found that rural high-income households often have higher consumption levels and more excellent consumption elasticity, making them more influenced by policies. This indicates that the consumption levels of rural low-income households remain relatively low, with a focus on essential consumption. The impact of the integration of medical insurance on the consumption of such households is still limited. The government and society should provide more policy support to low-income older adult households to improve their consumption levels.

The existing literature has yet to explore the mechanism through how integrating health insurance for urban and rural residents affects rural household consumption. This study primarily analyzes the mechanism based on the proportion of medical expenditure, health, and life expectancy, providing necessary complementary research. This study shows that integrating health insurance for urban–rural residents improve the consumption level by reducing the medical economic burden and improving health and life expectations. Firstly, rural older adult households are highly susceptible to economic and health risks, with a heavy burden of medical expenses. Integrating health insurance for urban and rural residents has improved the medical benefits for rural households, reducing the economic burden caused by health risks. This is beneficial for enhancing their consumption capacity. Secondly, integrating health insurance systems has reduced social risks for rural households, improved life expectancy, and increased consumer confidence. This finding is consistent with the research conclusions of Ma and Li (14). Lastly, this study indicates that the impact of this effect on survival consumption is higher than on total consumption. This suggests that the consumption structure of rural residents remains relatively low, thereby limiting their quality of life. Integrating health insurance for urban and rural residents plays a positive role in both objective and subjective factors of rural household consumption. Government-led institutional integration has positive policy effects and enhances rural household consumption.

## Conclusion and policy implications

This study systematically explores the impact of integrating health insurance for urban and rural residents on household consumption in rural areas by employing the DID and PSM-DID methods. The study uses panel data from the China Health and Retirement Longitudinal Study (CHARLS) conducted from 2013 to 2018. The research conclusions are as follows: Firstly, integrating urban–rural residents’ health insurance is associated with an increase in the survival consumption and total consumption of rural households. This conclusion remains valid after endogeneity and placebo tests. Secondly, the results also reveal heterogeneity, indicating that integrating urban and rural residents’ health insurance on the consumption of rural middle-aged, female, and high-income households is more significant for survival and total consumption. Thirdly, integrating urban and rural residents’ health insurance increases the consumption of rural older adult households by reducing their medical burden and improving their health and life expectancy. Generally, the mediating effect on survival consumption is more significant than on total consumption.

This paper provides the following policy implications: Firstly, The government should further deepen the equity of the primary health insurance system. Integrating health insurance for urban and rural residents is essential to enhancing institutional equity and has positive policy effects. The government should enhance the institutional integration of healthcare coverage for urban and rural residents while relieving the insurance premium burden on older adult and low-income families in rural areas. On this basis, it is necessary further to deepen the reform of the medical and health system and promote the phased coordination of fundamental healthcare coverage at the provincial and national levels to narrow the disparity in accessing medical and health services between urban and rural areas, stimulate rural development-oriented consumption, and achieve common prosperity. Secondly, it is imperative to improve the convenience of medical services for rural older adult households and fully leverage the positive consumption externalities of medical security. This study demonstrates that integrating health insurance systems has enhanced the consumption level of older adult households in rural areas. Therefore, the government should coordinate medical and healthcare resources in rural areas and actively steer medical service facilities, funds, and talents to focus on rural and remote areas, helping to improve the expectations of consumers and the quality of life of rural older adult households. Thirdly, it is imperative to address the medical service needs of rural women, families with low incomes, and older adult groups. This study suggests that the positive impact of integrating health insurance for urban and rural residents on vulnerable groups, such as low-income populations in rural areas still needs to be improved. Therefore, it is crucial to prioritize improving the quality of life for these groups. Establishing and improving healthcare and life assistance programs for vulnerable groups in rural areas provides convenient and targeted medical services, mitigates the negative impact of health risks on the lives of target groups, and enhances confidence in consumption for these families. Fourthly, Income is one of the most critical factors influencing consumption. Therefore, the government should increase investment in infrastructure construction in rural areas, such as water, electricity, roads, and the Internet, and expand the scale of family-based farming and private income sources in rural areas.

## Data availability statement

The original contributions presented in the study are included in the article/supplementary material, further inquiries can be directed to the corresponding authors.

## Ethics statement

The studies involving humans were approved by Ethical review and approval were waived for this study, due to the data used in this article coming from the public database, with which all subjects involved are anonymous. The studies were conducted in accordance with the local legislation and institutional requirements. The participants provided their written informed consent to participate in this study. Written informed consent was obtained from the individual(s) for the publication of any potentially identifiable images or data included in this article.

## Author contributions

JW: Conceptualization, Formal analysis, Writing – original draft. QH: Data curation, Methodology, Writing – review & editing.
